# Electron-Beam Writing of Atomic-Scale Reconstructions at Oxide Interfaces

**DOI:** 10.1021/acs.nanolett.4c02913

**Published:** 2024-11-01

**Authors:** Greta Segantini, Chih-Ying Hsu, Carl Willem Rischau, Patrick Blah, Mattias Matthiesen, Stefano Gariglio, Jean-Marc Triscone, Duncan T. L. Alexander, Andrea D. Caviglia

**Affiliations:** † Department of Quantum Matter Physics, University of Geneva, 24 Quai Ernest-Ansermet, CH-1211 Geneva 4, Switzerland; ‡ Electron Spectrometry and Microscopy Laboratory (LSME), Institute of Physics (IPHYS), Ecole Polytechnique Fédérale de Lausanne (EPFL), CH-1015 Lausanne, Switzerland; ¶ Kavli Institute of Nanoscience, Delft University of Technology, 2628 CJ Delft, The Netherlands

**Keywords:** oxide membranes, perovskites, interface, ionic bonding, in-situ e-beam writing

## Abstract

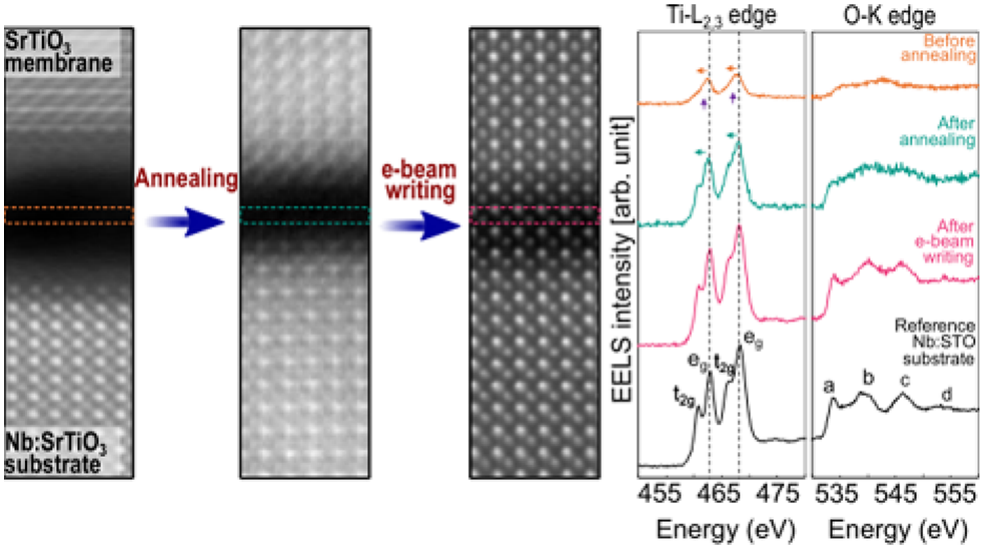

The epitaxial growth of complex oxides enables the production of high-quality films, yet substrate choice is restricted to certain symmetry and lattice parameters, thereby limiting the technological applications of epitaxial oxides. In comparison, the development of free-standing oxide membranes gives opportunities to create novel heterostructures by nonepitaxial stacking of membranes, opening new possibilities for materials design. Here, we introduce a method for writing, with atomic precision, ionically bonded crystalline materials across the gap between an oxide membrane and a carrier substrate. The process involves a thermal pretreatment, followed by localized exposure to the raster scan of a scanning transmission electron microscopy (STEM) beam. STEM imaging and electron energy-loss spectroscopy show that we achieve atomically sharp interface reconstructions between a 30-nm-thick SrTiO_3_ membrane and a niobium-doped SrTiO_3_(001)-oriented carrier substrate. These findings indicate new strategies for fabricating synthetic heterostructures with novel structural and electronic properties.

Complex oxides exhibit a broad spectrum of functionalities, including ferroelectricity, ferromagnetism, and high-temperature superconductivity.^1,2^ In recent years, significant attention has been directed toward their potential applications across various technological domains.^3−5^ Epitaxial growth enables the fabrication of high-quality oxide films, providing an ideal platform for investigating their physical properties at the atomic level. Moreover, interface engineering of epitaxially grown oxide layers led to the discovery of intriguing interface phenomena.^6−8^ However, the epitaxial relationship between the thin film and substrate imposes limitations on the application of stimuli to the oxides, such as strain, and confines the substrate selection to those meeting specific symmetry and lattice spacing requirements. Inspired by the isolation of 2D materials, such as graphene and transition-metal dichalchogenides, a promising way to overcome intrinsic limitations of epitaxial oxides is to detach them from their growth substrate. Among the strategies explored, the chemical lift-off approach has gained considerable interest.^9−11^ In this approach, epitaxially grown sacrificial layers are dissolved using suitable etchants, thereby releasing oxide layers as membranes that can be transferred and stacked, free of epitaxial restrictions. Literature reports demonstrated the remarkable response of oxide membranes to strain,^12−14^ and the ability to control the twist angle of stacked membranes to create and manipulate moiré patterns.^10,15^ These systems hold promise for applications in nanoelectronics, including nonvolatile memories, sensors, and flexible electronics.^16−18^ While research on oxide membranes has yielded innovative results, the ability to create a strong chemical bond between the membrane and a carrier substrate (or second membrane) onto which it is transferred remains relatively unexplored.

Here, we report the controlled formation of interfacial ionic bonds between a 30-nm-thick SrTiO_3_ membrane and a niobium-doped SrTiO_3_(001)-oriented (Nb:SrTiO_3_) carrier substrate. The SrTiO_3_ membranes were fabricated by epitaxial growth of a 15-nm-thick Sr_3_Al_2_O_6_ sacrificial layer followed by a 30-nm-thick SrTiO_3_ layer on a SrTiO_3_(001)-oriented substrate using pulsed-laser deposition (PLD). As schematically illustrated in Figure 1, a strip of poly(dimethylsiloxane) (PDMS) was applied to cover the entire surface of the SrTiO_3_ layer for the lift-off process, and the structure was immersed in deionized water at room temperature to dissolve the Sr_3_Al_2_O_6_ layer. The resulting SrTiO_3_ membrane was then transferred onto a Nb:SrTiO_3_(001)-oriented nonterminated substrate, and PDMS removed. The Supporting Information (SI) provides details on the transfer procedure along with other experimental parameters. Figure 1 also shows high-angle annular dark-field (HAADF) STEM images of sample cross sections: SrTiO_3_(001)/Sr_3_Al_2_O_6_/SrTiO_3_ heterostructure before lift-off (left), and SrTiO_3_ membrane after transfer (right). Combined with the X-ray diffraction patterns of SI Figure S1, these images show that the good crystalline quality of the SrTiO_3_ membrane is preserved during transfer. We note that the initially flat layer does sometimes acquire some low amplitude modulations after transfer, as measured using atomic force microscopy (SI, Figure S2). In the following, three samples are studied. One is as-transferred (Sample 0), while the other two underwent an additional thermal annealing step at atmospheric pressure for 1 h, at temperatures of 550 °C (Sample 550) or 750 °C (Sample 750).

**Figure 1:**
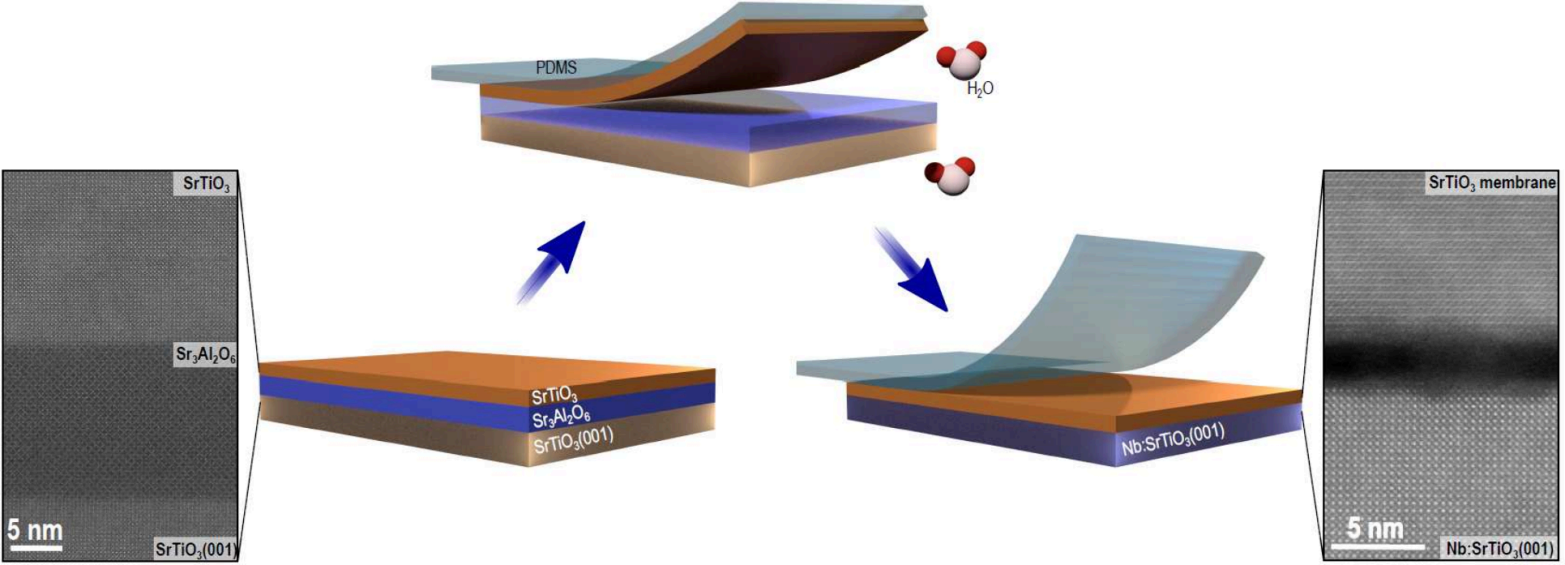
Schematic of the membrane fabrication process: The Sr_3_Al_2_O_6_ sacrificial layer and the SrTiO_3_ membrane were synthesized using PLD. Subsequently, a PDMS sheet was applied to the surface of the SrTiO_3_ layer, and the entire structure was immersed in deionized water. Following the dissolution of the Sr_3_Al_2_O_6_ layer, the resulting SrTiO_3_ membrane was transferred onto the Nb:SrTiO_3_(001) substrate. HAADF STEM cross-sectional images of the heterostructure SrTiO_3_(001)/Sr_3_Al_2_O_6_/SrTiO_3_ before lift-off and of the SrTiO_3_ membrane transferred onto the Nb:SrTiO_3_(001) substrate are shown on the left and on the right, respectively. In the latter, an interface gap of ∼2 nm between the SrTiO_3_ membrane and the Nb:SrTiO_3_(001) substrate is distinguishable.

First, we examine the effect of annealing on the substrate/membrane system. Outside of any height-modulated membrane regions, the adjacent crystalline surfaces of the Nb:SrTiO_3_(001) substrate and SrTiO_3_ membrane are relatively smooth and uniformly spaced, with a gap between them that evolves under thermal annealing (see low magnification cross-sectional STEM images in SI Figure S3). In Sample 0, the gap measures ∼2 nm in width. The origin of the gap is associated with the presence of contamination species on the substrate surface and on the membrane surface in contact with the Sr_3_Al_2_O_6_ sacrificial layer, stemming from its dissolution in deionized water.^19^Figure 2a shows a higher magnification image of the interface gap in Sample 0. Given that the intensity of an HAADF image *I* ∝ *Z*^1.6–1.9^ (average atomic number *Z*),^20^ the dark contrast of the gap is attributed to its amorphous, disordered nature and its lower density compared to the crystalline material either side. Energy dispersive X-ray spectroscopy (EDXS) was used to analyze the elements present within the gap. Major elements of strontium, titanium and oxygen were found, together with carbon, a common contaminant from air exposure, and calcium and aluminum, which are residuals from the lift-off process (SI Figure S4). Further, electron energy-loss spectroscopy (EELS) was used to study the electronic/bonding state of the Ti and O going across the gap from the substrate to the membrane. As shown in Figure 2a, the Ti *L*-edge of the crystalline substrate (spectrum #1) presents the signature splitting of Ti *L*_2_ and *L*_3_ peaks. This results from spin–orbit coupling, which gives rise to two distinct peaks that are attributed to the *t*_2*g*_ and *e*_*g*_ molecular orbitals, characteristic of the Ti^4+^ in octahedral symmetry.^21,22^ The O *K*-edge in turn presents a series of well-defined peaks (labeled a, b, c, and d) that are characteristic of SrTiO_3_.^22^ As expected from its high-quality crystalline nature, spectrum #5 from the membrane shows features equivalent to those of the Nb:SrTiO_3_(001) substrate. However, spectrum #3 from the middle of the gap is distinctly different. Owing to the lower density of material, the edge intensities are strongly reduced. Further, no splitting is visible in the Ti *L*_2,3_ peaks, which are also left-shifted by ∼1 eV. Equally, the first peak of the *O* K-edge is shifted to a higher energy. These observations are consistent with a Ti valence in the gap of ∼ Ti^2+^, and a loss of octahedral coordination with oxygen atoms.^23^ We note that, despite spectra #2 and #4 being extracted close to the interface, they still display the same characteristics as those obtained from the substrate and membrane.

**Figure 2:**
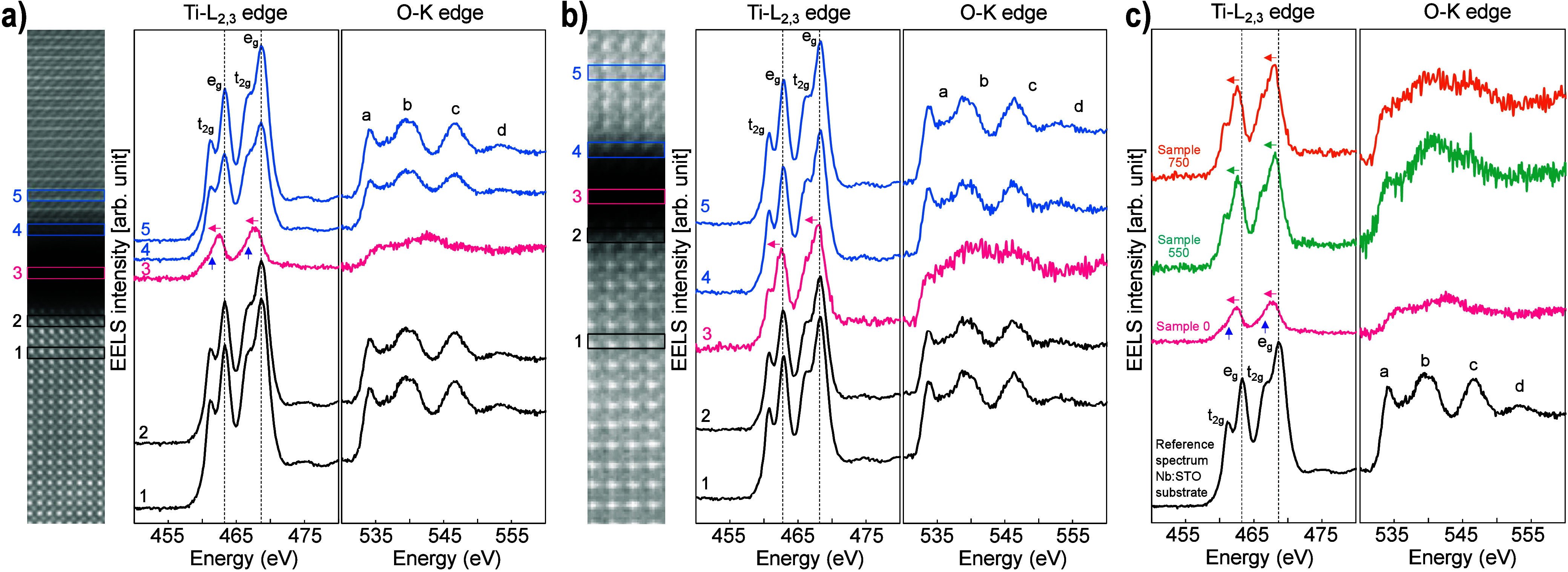
Effect of annealing of the SrTiO_3_ membrane on Nb:SrTiO_3_(001) substrate. (a) STEM-EELS analysis of Sample 0. From left to right: HAADF cross-sectional image of Nb:SrTiO_3_(001) substrate/SrTiO_3_ membrane, background-subtracted Ti-*L*_2,3_ and O-*K* edges extracted from #1 Nb:SrTiO_3_(001) substrate, #2 Nb:SrTiO_3_(001) substrate near the bottom interface, #3 center of the gap, #4 SrTiO_3_ membrane near the top interface, and #5 SrTiO_3_ membrane. The Ti-*L*_2,3_ and O-*K* edges in spectrum #3 reveal a clear change compared to the crystalline SrTiO_3_. In particular, the splitting of the Ti *L*_3_ and *L*_2_ peaks observed in spectra #1, #2, #4, and #5, that indicates a Ti^4+^ oxidation state, is no longer visible, suggesting a change in Ti valence from 4+ to 2+ . (b) STEM-EELS analysis of Sample 750: spectrum #3 of Ti-*L*_2,3_ edge indicates that Ti has shifted toward 4+ valence; the first fine structure peak of the O-*K* edge has also moved to a lower energy compared to gap spectrum #3 in (a). (c) Comparison of Ti-*L*_2,3_ and O-*K* edges obtained from Sample 0, Sample 550, and Sample 750 extracted in the center of the gap together with a reference from the Nb:SrTiO_3_(001) substrate. The evolution of the Ti-*L*_2,3_ edges as a function of the annealing temperature demonstrates a clear change in Ti valence state. All the displayed spectra are background-subtracted, equivalently normalized by substrate intensities, and aligned on the energy-loss axis using the O-*K* edge onset energy (532 eV). Note that, for compactness, the HAADF images are cropped from the full width of the original mapped areas. The EEL spectra are integrated from the full map width.

Figure 2b shows the STEM-EELS analysis for Sample 750 (see SI Figure S5 for Sample 550). The thermal annealing induces a number of changes in the gap. First, its width decreases to ∼0.9 nm. At the same time, the normalized intensities of the Ti and O edges within the gap are more than doubled compared to those in Sample 0. Together, these imply a densification from annealing without loss of material content. Figure 2b spectrum #3 from the gap also shows that the ionization edge structures are modified. Both the Ti *L*_2_ and *L*_3_ peaks present a discernible splitting, with a reduced left shift, and the first peak of the O *K*-edge shifts toward the edge onset. Both these results suggest that annealing has moved the Ti valence state up from Ti^2+^ toward Ti^4+^.

To profile the spectral evolution from annealing, Figure 2c shows EEL spectra extracted from the interface gaps of samples 0, 550, and 750, together with a reference spectrum from the Nb:SrTiO_3_(001) substrate. Each spectrum is normalized in intensity by its data set’s substrate spectrum and aligned by the onset energy of the O *K*-edge at 532 eV. The figure underscores the onset of splitting and reduced left shift of the Ti *L*_2_ and *L*_3_ peaks with thermal annealing. Also, the O *K*-edge transitions toward having features similar to those of the substrate reference spectrum. Overall, therefore, the STEM-EELS analyses show that the annealing procedure not only reduces gap width between membrane and Nb:SrTiO_3_(001) substrate but also modifies the Ti valence state at the interface from Ti^2+^ toward Ti^4+^.

Finally, we point out the in-plane structural misalignment observed between the membrane and the substrate in the HAADF cross-sectional images of Figures 2a and b, which were both acquired along a reference zone axis of the substrate. As the membranes are not in zone axis, their atomic columns cannot be (clearly) distinguished. From tilting the sample stage, this misalignment was quantified to be ∼2° for Sample 0 and ∼0.7° for Sample 750, and it is considered a natural consequence of applying a small twist during the manual transfer process.

In the second part of this study, we look at the impact of the STEM electron-beam (“e-beam”) on the substrate/membrane interface. The data presented in the previous section were taken using acquisition conditions that were carefully tuned in order to measure the three samples in their original condition (see SI Table S1). However, we observed that when the e-beam flux is above a certain threshold value (discussed below), rastering it across the ∼0.9 nm interface gap of an annealed sample leads to its structural modification. Figure 3 illustrates this structural evolution. Each row presents frames from the HAADF STEM image series of the three samples that were acquired under “STEM-EDXS” conditions (300 kV high tension, 2 μs dwell time, 250 pA beam current, multiple frame series). In Figure 3a for Sample 0 no change is observed within the gap, even after 800 frames. In contrast, in Figure 3b, c for Samples 550 and 750, it is evident that, under the 250 pA e-beam raster scan, a crystal structure forms within the gap. For Sample 550, a new atomic structure within the gap first becomes visible after 250 frames, corresponding to a cumulative electron dose of ∼3.04 × 10^6^ e^–^ Å^–2^. Seemingly, it propagates from the substrate toward the membrane, as indicated by the orange arrows. By frame 500 (electron dose ∼6.08 × 10^6^ e^–^ Å^–2^), the crystalline structure bridges the full gap, as marked by two orange arrows. Note that because the membrane is twisted ∼2° relative to the substrate, it is misaligned for atomic column imaging and only shows horizontal lattice planes in the images.

**Figure 3:**
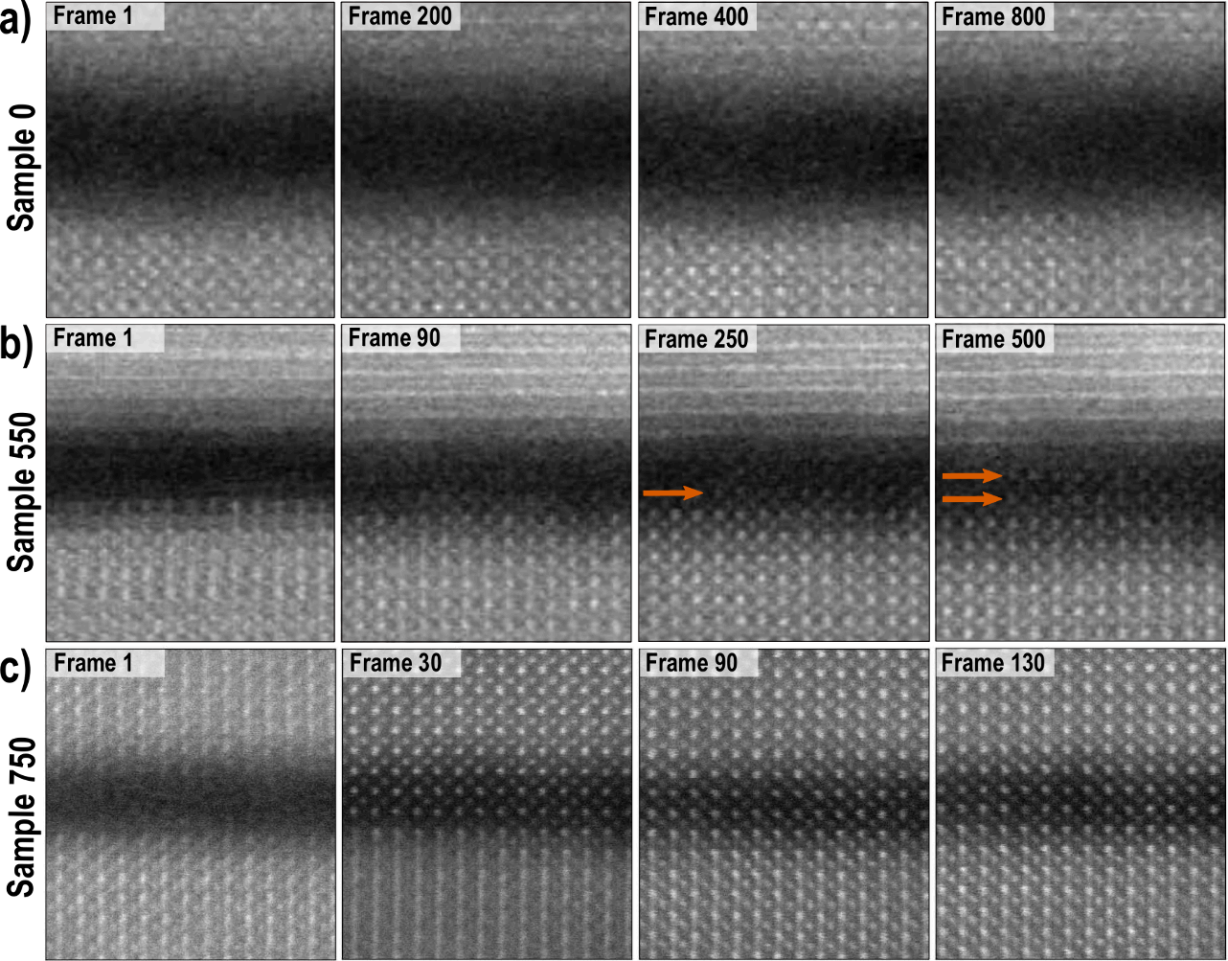
Effect of STEM-EDXS raster scan conditions on Nb:SrTiO_3_(001) substrate/SrTiO_3_ membrane system, showing the evolution of the interface gap as a function of the acquired number of frames. (a) Sample 0: no evident changes are observed after 800 frames. (b) Sample 550, ordered atomic structure emerges within the gap after 250 frames. At the final frame 500, the gap is largely filled with crystalline structure. (c) Sample 750, crystal structure completely fills the gap after 30 frames. By frame 130, the cation sites of substrate and membrane have also come into alignment.

Figure 3c portrays the same analysis for Sample 750, where a new crystalline structure has formed across the whole gap after just 30 frames, corresponding to an electron dose of ∼2.92 × 10^6^ e^–^ Å^–2^. Remarkably, structural transformation continues; by frame 130 the interface region is fully reconstructed, with the image showing that, either side of the crystallized gap, the cation sites of membrane and substrate have themselves come into alignment. SI Figure S6 shows the accompanying atomic resolution EDXS elemental maps, integrated from the full series of mapping frames, where the net count line profiles confirm that the brighter and darker cation rows across the interface region respectively correspond to Sr and Ti.

In the case of Sample 750, the new crystal structure appears to propagate from the membrane toward the substrate, in contrast with Figure 3b for Sample 550. After repeated image series acquisitions of different regions under the same EDXS mapping parameters but different sample tilts, we find that these opposing observations are mostly a consequence of the chosen imaging condition. In fact, we conclude that the crystal structure that forms within the gap originates from both sides. However, our observation of structural propagation is sensitive to the alignment of the crystal structure to the incident e-beam; atomic columns are much more distinct when the crystal is aligned very close to a perfect zone axis condition (i.e., a condition with strong electron channeling down the atomic columns). Therefore, when the substrate is better aligned to the incident beam, as in Figure 3b, the new structure appears to propagate from the substrate; when instead, the membrane is better aligned, it appears to start from the membrane (Figure 3c).

In Figure 4 we study these bridging crystalline structures using EELS, in the case of Sample 750. Figure 4a shows the Ti-*L* and the O-*K* edges at the same region where the EDXS scanning of Figure 3c was made. Unlike the as-annealed condition of Figure 2b, the Ti-*L*_2,3_ and O-*K* edges at the center of the interface region now closely resemble those of the membrane and Nb:SrTiO_3_(001) substrate. This indicates the formation of the SrTiO_3_ crystal structure, such that ionic bonds have formed across the interface gap. (To help illustrate the evolution in the EELS fine structure, SI Figure S7 presents EEL spectra projected along a line in the out-of-plane direction from before and after the local e-beam irradiation.)

**Figure 4:**
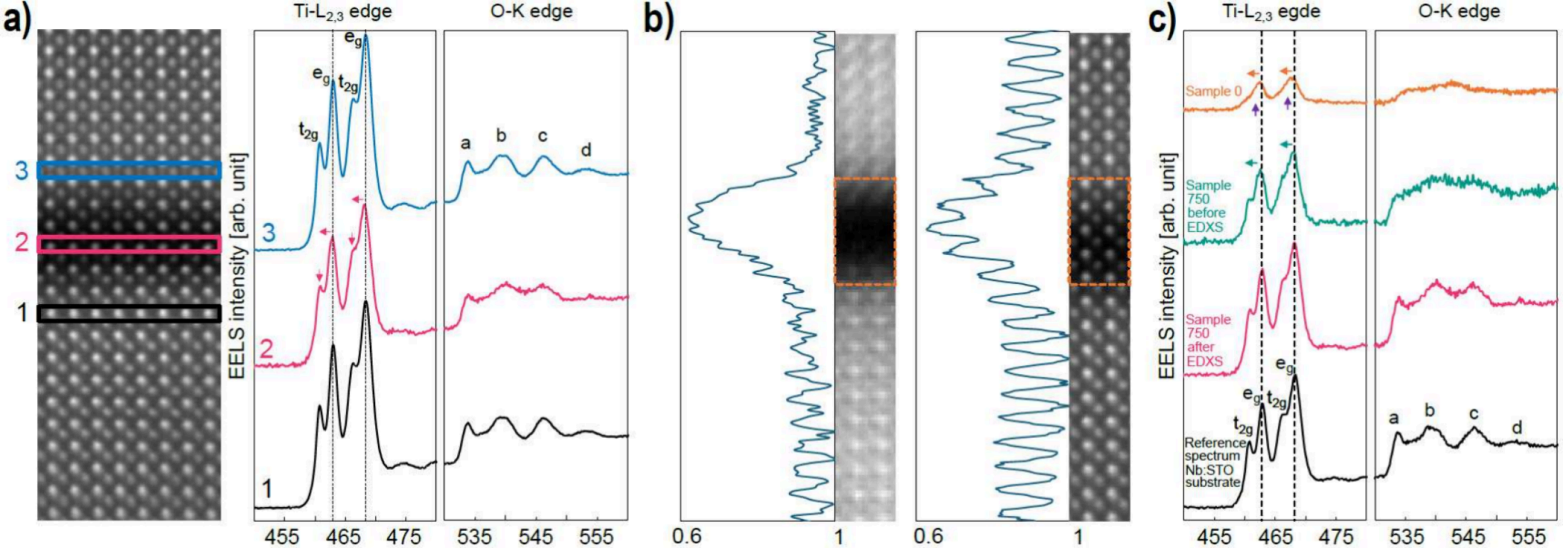
Effect of the EDXS condition e-beam raster scan on Sample 750. (a) STEM-EELS analysis after the EDXS raster scanning that is shown in Figure 3c. From left to right, the HAADF image, Ti-*L*_2,3_ edge, and O-*K* edges acquired from the Nb:SrTiO_3_(001) substrate, the gap, and the membrane. The HAADF image depicts clear atomic columns within the gap. EEL spectral features of the Ti-*L*_2,3_ and O-*K* edges closely resemble those observed in the Nb:SrTiO_3_(001) substrate and membrane. (b) Ti-*L* edge integrated signal from EELS map acquired from the initial “pristine” area, and from the same area after the EDXS raster scanning shown in Figure 3c. The consistent intensity of the Ti integrated signal indicates no mass loss or gain of Ti atoms within the gap during the structural reorganization to a crystalline structure. The HAADF images are cropped from the full width of the original mapped areas. (c) Comparison of EEL spectra from the center of the interface gap for as-transferred, 750 °C annealed, and 750 °C annealed–EDXS raster scanned, together with a reference spectrum from the Nb:SrTiO_3_(001) substrate. Displayed EEL spectra were processed as for Figure 2.

In Figure 4b, we consider mass preservation during the e-beam induced restructuring. HAADF images from before and after EDXS scanning show that after EDXS scanning clear atom columns are visible across the gap (marked in orange boxes). Next to the HAADF images, we plot respective line-profiles of the Ti-*L* edge integrated signal. While in the “after” case, the line profile acquires strong modulations corresponding to the new, well-defined atomic planes, the overall Ti signal intensity remains unchanged. In both cases, it shows a drop of ∼40% at the gap center compared to that of the substrate/membrane. This confirms, on the one hand, the less dense nature of the gap compared to the substrate and membrane and, on the other hand, that the crystal structure observed after EDXS scanning derives from the reorganization of existing Ti cations into octahedral coordination with oxygen atoms, without incorporating extra Ti cations from elsewhere.

Figure 4c provides a summary of spectral progression across sample processing, showing gap EEL spectra for the as-transferred (orange), 750 °C-annealed (blue-green), and 750 °C-annealed+raster-scanned conditions (pink). Only in the latter case do the Ti-*L*_2,3_ and the O-*K* edge features align with those of the reference spectrum from the bulk substrate (black), showing a Ti valence state modification from Ti^2+^ to Ti^4+^. With a combination of annealing and e-beam raster scanning, the originally amorphous gap has been bridged by ionic bonds between the membrane and the Nb:SrTiO_3_(001) substrate, complemented by octahedral coordination of the residual oxygen atoms.

We now consider the mechanisms behind the e-beam induced writing of atomic structure. The powerful scope for using the STEM analytical probe to create and tailor structures down to the atomic scale is generally established.^24^ Further, a recrystallization of ion-beam amorphized SrTiO_3_ on SrTiO_3_ single crystal under e-beam raster scanning, into a perfect, epitaxial SrTiO_3_ crystal lattice, was previously observed by Jesse et al.^25^ Sample damage or modification by an incident e-beam is typically ascribed to one of two basic possibilities: first, ballistic interaction of the fast transmitting electron with an atomic nucleus that leads to atomic displacement (knock-on damage); second, excitation of an atomic electron above the Fermi level, temporarily leaving a hole that may destabilize the atom’s bonding, which then leads to a change in atomic bonding and structure (radiolysis).^26^ Jesse et al. hypothesized that the crystallization of amorphous SrTiO_3_ was promoted by knock-on damage.^25^ While knock-on often sputters material, we assume that it was instead conceived to locally rearrange atomic species without mass loss. We, however, hypothesize that in our case radiolysis is the critical factor, as also concluded for e-beam restructuring of rutile TiO_2_.^27^ Radiolysis electronically excites the atoms in the interface gap. When these atoms recover to a ground state, they go to a new, more stable state as they rearrange to form the observed ionically bonded crystal structure. Potentially, the ionization of Ti cations directly facilitates the transfer of their charge to O anions, leading to the formation of ionic bonds. In support of our hypothesis, we identify an upper bound of electron flux of ∼10^10^ e^–^ Å^–2^ s^–1^ that avoids crystallization of the gap region for Sample 750, as exemplified for EDXS in SI Figure S8. (See the SI section E-Beam Flux Effects for details.) This is hard to understand in the context of pure knock-on, where damage is perceived as being permanent and hence proportional to e-beam dose and nonrecoverable under any flux. Knock-on damage is also typically associated with mass-loss from sputtering, which we do not observe (Figure 4b). However, the threshold is consistent with radiolysis, when atoms are allowed to recover to their initial ground state under sufficiently low flux. To emphasize the critical nature of this electron flux, when using a 0.25 Å step-size, increasing the e-beam current used for the EELS mapping from 90 to 100 pA was enough to induce observable atomic rearrangements within the gap during multiple pass acquisitions. An analogous nature of flux thresholds has been observed for preserving the pristine O sublattice of crystalline samples of cuprates and nickelates.^28,29^

As mentioned earlier, continued exposure to the e-beam raster scan not only produces crystalline structure bridging the gap but can realign adjacent zones of substrate and membrane that are also exposed. In Figure 3c this leads to the local “untwisting” of membrane and substrate to the same zone axis orientation, producing the high quality lattice spanning across them in Figure 4a. Since the surrounding bulk lattices of both the substrate and membrane remain unaffected, these local displacements must be accommodated by strong local lattice distortions or defect creation. As such distortions are primarily in-plane, it is, however, difficult to discern them using cross-sectional imaging. Wang et al. interpreted the structural rotation of the first two monolayers of an SrTiO_3_ membrane, which had been bonded to a sapphire substrate, by subjecting them to a 1000 °C laser-induced, ultrahigh vacuum thermal anneal.^19^ To give a comparative indication of the possible zone of distortion associated with the e-beam writing of crystalline structure here, SI Figure S9 presents lower magnification HAADF STEM images of Sample 750 before and after a local e-beam raster scan (this time made using a “focus window”).

After exposure, crystalline lattice 7–10 unit cells deep into the substrate or membrane have twisted into a new configuration that differs from the unexposed surrounding area. In this case, the two pre-existing crystals appear to have become less aligned during the reconfiguration. However, in our interpretation, we cannot control for the effects of translational displacements or membrane subgrain boundaries that are invisible in the projection of the STEM image. Nevertheless, it is clear that the structurally affected zone penetrates far from the interface, implying a strong effect of bonding across the gap that is consistent with the formation of ionic bonds. Such strong effects even extend to another 750 °C annealed sample having a larger membrane/substrate misalignment of ∼4°, which shows both full gap reconstruction and ∼3 unit cell substrate realignment after sufficient EDXS raster scanning (SI Figure S10). Finally, we point out that within the gap the new crystalline structure can extend a couple of unit cells laterally beyond the region directly impacted by the raster scan (red box in SI Figure S9b). This suggests that incoherently scattered secondary electrons, or coherent excitations with longer interaction lengths (plasmons, phonons), may also play a role in the crystallization,^26,30^ hinting at a complexity of interactions that needs further investigation to understand fully.

In summary, we demonstrate the local writing of crystalline structure across the interface gap between a 30-nm-thick SrTiO_3_ membrane and a Nb:SrTiO_3_ (001)-oriented substrate, achieved through two steps. First, thermal annealing of the bulk sample reduces the gap width and shifts the valency of the residual Ti and O atoms in the gap from TiO equivalents to more oxidized species. Second, a STEM e-beam raster scan induces the Sr, Ti and O atoms in the interface gap to rearrange into an ionically bonded crystalline lattice. The results indicate that the annealing temperature strongly influences the efficacy of interface reconstruction by the e-beam raster scan, with a slower process observed for the sample annealed at 550 °C compared to that annealed at 750 °C. We note that, in an extra sample that was annealed for 3 h at 750 °C, and that contained bumps in the transferred membrane, it was possible to crystallize across gaps of ∼4 nm in width. This implies that the shift in Ti valence from 2+ toward 4+ (and O valence modification) induced by thermal annealing (Figure 2) is the crucial factor for the e-beam-induced crystallization to occur, rather than the reduction in gap size. This importance of the initial electronic state is further consistent with our hypothesis that radiolysis is the primary driver of the interface reconstruction.

The method introduced here allows nanometric precision in the crystal structure writing. For each annealed condition, the extent of structural transformation can be controlled by tuning a combination of electron flux and total dose, with the possibility of completely avoiding reconstruction by staying below threshold flux values. With sufficient flux and dose, structural effects can propagate up to ∼10 unit cells deep into the substrate and membrane, creating localized lattice strains.

This precise control of interface reconstruction between perovskite oxides therefore represents a powerful tool for discovering new interfacial phenomena, for instance, by purposefully inducing strain gradients. These could be tailored, for instance, by choice of twist angle or interfacing materials with different lattice parameters to create local tensile or compressive strain. Further, employing other electron probes, such as scanning electron microscopy (SEM) e-beams or e-beam lithography, could enable interface reconstruction over large areas within the membrane/substrate system. We note that, since ionization cross section increases as beam energy decreases, a radiolysis-driven reconstruction is fully compatible with the use of SEM. Our method thus suggests an alternative pathway for creating synthetic oxide membrane-based heterostructures, with the ability to selectively induce ionic bonding between them.

## Supporting Information Available

Experimental methods; E-beam flux effects; X-ray diffraction patterns; Atomic force microscopy results; Large field of view STEM images of the 3 samples; STEM-EELS of Sample 550; STEM-EDXS of Sample 0 and 750; Projected EEL spectra from before and after irradiation of Sample 750; HAADF STEM images and frames from other raster scan/sample conditions. (PDF)
