# Supplementary material for: Electron-Beam Writing of Atomic-Scale Reconstructions at Oxide Interfaces
Source: Nano Lett. 2024 Nov 1;24(45):14191–7. doi: 10.1021/acs.nanolett.4c02913 (PMC11565737; doi:10.1021/acs.nanolett.4c02913)
Supplement: Supplementary file 1 — nl4c02913_si_001.pdf [file nl4c02913_si_001.pdf]

Supporting Information for: Electron-Beam Writing of Atomic-Scale Reconstructions at Oxide Interfaces

Greta Segantini,[†] Chih-Ying Hsu,[‡,†] Carl Willem Rischau,[†] Patrick Blah,[¶] Mattias Matthiesen,[¶] Stefano Gariglio,[†] Jean-Marc Triscone,[†] Duncan T.L. Alexander,[‡] and Andrea D. Caviglia*,[†]

†*Department of Quantum Matter Physics, University of Geneva, 24 Quai Ernest-Ansermet, CH-1211 Geneva 4, Switzerland.*

‡*Electron Spectrometry and Microscopy Laboratory (LSME), Institute of Physics (IPHYS), Ecole Polytechnique Fédérale de Lausanne (EPFL), CH-1015 Lausanne, Switzerland.*

¶*Kavli Institute of Nanoscience, Delft University of Technology, 2628 CJ Delft, The Netherlands.*

E-mail: greta.segantini@unige.ch

Experimental

Sample Preparation

The 30 nm-thick $SrTiO_3$ layer was epitaxially grown on a 15 nm-thick $Sr_3Al_2O_3$ sacrificial layer on a (001)-oriented non-terminated $SrTiO_3$ substrate. The films were grown using pulsed-laser deposition (PLD) equipped with a KrF excimer laser (λ = 248 nm). The $Sr_3Al_2O_6$ layer was deposited on the $SrTiO_3$(001) substrate using a laser fluency of 2.2

J.cm^{-2}, and the $SrTiO_3$ layer with a laser fluency of 1.6 J.cm^{-2}. Both layers were grown at 700 °C with an oxygen pressure of 10^{-5} mbar. Following the deposition, the sample was cooled down in the same oxygen pressure for 180 minutes. The lift-off procedure was performed by applying a polydimethylsiloxane (PDMS) strip to the $SrTiO_3$ surface, followed by transferring the structure to de-ionized water for approximately 1 h to dissolve the $Sr_3Al_2O_6$ sacrificial layer. Subsequently, the PDMS with the resulting $SrTiO_3$ membrane was transferred onto the Nb:$SrTiO_3$(001) (0.5 wt%) non-terminated substrate. The transfer process consisted in placing the Nb:$SrTiO_3$(001) substrate on a hot plate maintained at 80 °C. Once the PDMS/membrane system adhered to the substrate, it was pressed down for 90 s. The annealing procedure was performed in a tubular furnace kept at atmospheric pressure.

Atomic Force Microscopy

The surface morphology analysis was conducted using AFM with a *Digital Instrument* Nanoscope Multimode DI4 with a *Nanonis* controller.

X-Ray Diffraction

XRD measurements were performed using a *Panalytical X'Pert* diffractometer with Cu Kα1 radiation (1.54 Å) equipped with a 2-bounce Ge(220) monochromator and a triple axis detector.

TEM Lamella Preparation

The TEM lamellae in this study were prepared by focused ion beam milling using a Zeiss NVision 40. The lamellae preparation follows a typical preparation procedure with milling beam energy of 30 keV and currents varying from 1.5 nA down to 80 pA. At the end of milling, each lamella was cleaned using a 5 keV beam with a current of 30 pA. The final thicknesses of the lamellae are estimated to be between approximately 40–70 nm according

to low-loss EELS analyses.

STEM Analyses

The STEM analyses were carried out on a double aberration-corrected FEI Titan Themis 80-300, using a 300 keV beam energy and 20 mrad probe convergence semi-angle. This tool is equipped with the four quadrant ChemiSTEM SuperX EDXS system, and a GATAN GIF Continuum ERS EEL spectrometer with K3 direct detection camera that was operated in counting mode. EDXS data were acquired using Thermo Fisher Scientific Velox software, from which HAADF image series are exported. The displayed EDXS datasets were recorded using a 250 pA beam current and a 2 μs dwell time. However, additional experiments were made using beam currents of 100 and 150 pA in order to investigate electron flux (i.e. dose rate) thresholds for sample modification.

EELS data were recorded using Gatan DigitalMicrograph 3.5, and with a semi-angle of collection on the spectrometer of 40.8 mrad. The EELS acquisition parameters were rigorously selected to avoid any beam-induced structural modification of the interface gap, as deduced from comparing HAADF images recorded before and after the EELS mapping. These parameters were: singleEELS mode with 0.34 ms dwell time per pixel (minimum possible dwell time for the K3 detector), and the combinations of e-beam current and pixel step-size listed in Table 1 below. These parameters also give near 100% duty cycle during spectral acquisition, therefore maximising the utilization of the incident electrons. Each map corresponds to 6 integrated frames. Active drift correction was applied between acquisition of each frame, with drift measured using a separate reference region. Table 1 also gives an example condition that showed evidence of structural changes during the dataset acquisition from an annealed sample (100 pA beam current, 0.25 Å step-size). This condition could however be safely applied for the data shown in Figure 4a, since that sample had already been modified by the prior EDXS mapping.

Table 1: EELS acquisition conditions of dataset shown for different samples.

Sample	Figure	Dwell time (ms)	Current (pA)	Step-size (Å)	# of frames
Sample 0	2a	3.4	100	0.5	6
Sample 550	S5	3.4	90	0.25	6
Sample 750	2b	3.4	90	0.5	6
750 after EDXS	4a	3.4	100	0.25	6
Sample modified		3.4	100	0.25	6

In terms of data processing, the EEL spectrum images were denoised using principal component analysis with the standard MSA function available in DigitalMicrograph 3.5.[1] From the denoised datacubes, pixel integrated spectra were extracted, and spectral background removed using a standard power-law fitting. The intensity scales of displayed spectra are normalized by the Nb:$SrTiO_3$(001) substrate spectrum of each dataset, and calibrated on the energy axis by the O-K edge onset energy (532 eV) in the substrate, to facilitate the comparison between different datasets. We note that deconvolution of multiple scattering was not applied, because the increased electron flux of dualEELS mode was incompatible with avoiding beam-induced structural changes.

E-Beam Flux Effects

As detailed in Experimental, the effects of different e-beam currents on the annealed $SrTiO_3$ membrane/Nb:$SrTiO_3$(001) substrate samples were tested, for both EDXS (2 μs dwell time; 100, 150 and 250 pA) and EELS (0.34 ms dwell time; 90 and 100 pA) mapping acquisition conditions, combined with various pixel step-sizes.

In the EDXS case, no structural evolution of the interface gap of Sample 750 was observed when applying the lowest e-beam current of 100 pA, as demonstrated by the series of HAADF image frames in SI Figure S8. Applying a current of 150 pA instead led to slow structural changes. Under EELS conditions, the equivalent e-beam current limit for avoiding structural modification is 90 pA when using a 0.25 Å step-size. Notably, these limiting conditions *both* correspond to an electron flux in the region of $\sim 10^{10}$ e^{-} $Å^{-2}$ s^{-1}, even though pixel dwell

times were very different.

The EDXS tests at lower beam currents were made on a fourth sample that was annealed at 750 °C for 3 h. This latter sample was also employed to study the effect of the misalignment between the membrane and substrate on the interface reconstruction, which in this case was estimated to be $\sim 4°$. SI Figure S10 shows image series frames when applying our "standard" EDXS conditions of 250 pA beam current (electron flux $\sim 2.4 \times 10^{10}$ e^- $Å^{-2}$ s^{-1}). Even with this larger misalignment, crystal structure fills the ~ 0.9 nm gap within the first 90 frames. This observation implies that, even at larger misalignment angles, an ionic bond is formed between the membrane and substrate. With continued e-beam raster scanning, the misalignment between the membrane and the substrate is also corrected. However, in this case, only the misalignment of the first 3 unit cells of the substrate was corrected, rather than the deeper propagation of alignment seen in Figure 3c.

Supporting Information Figures

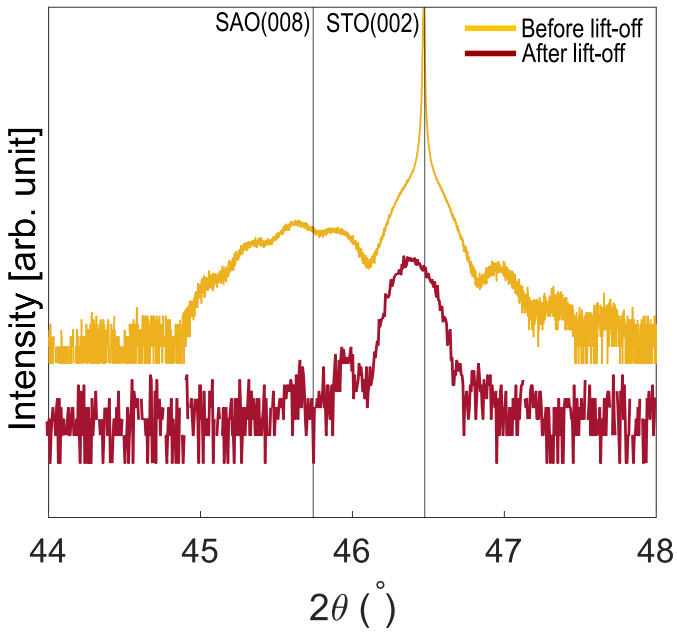

Figure S1: X-ray diffraction patterns of the $SrTiO_3(001)/Sr_3Al_2O_6/SrTiO_3$ heterostructure before lift-off (top curve), and of the $SrTiO_3$ membrane on PDMS after lift-off (bottom curve). The patterns show that the crystalline structure of the $SrTiO_3$ membrane is preserved after the lift-off procedure. Fitting the pattern of the lifted-off membrane on PDMS indicates a slight increase of the $SrTiO_3$ out-of-plane c lattice parameter, to a value of ∼3.91 Å

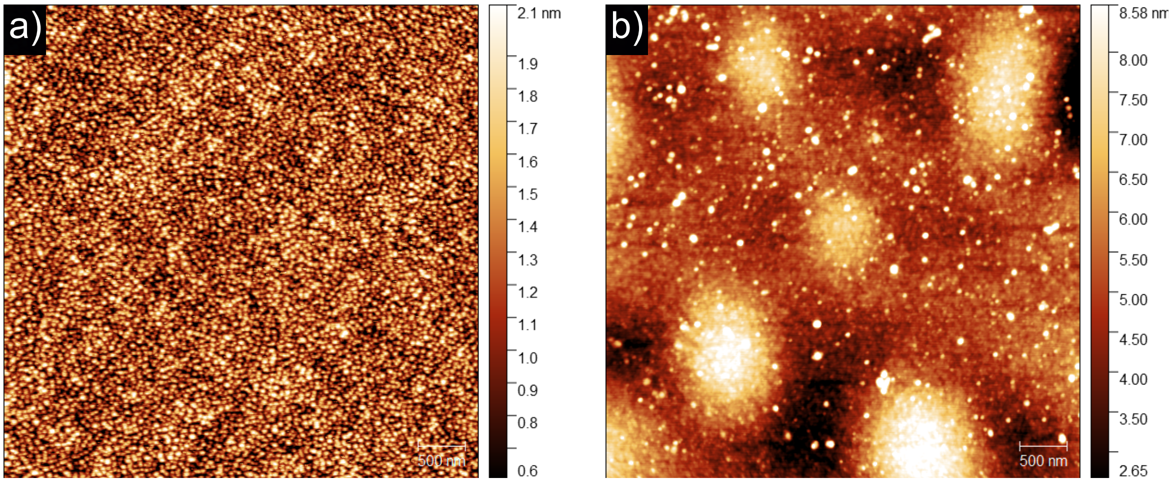

Figure S2: Surface topography acquired using atomic forces microscopy of: a) $SrTiO_3(001)/Sr_3Al_2O_6/SrTiO_3$ heterostructure before the lift-off procedure, with a root mean square (RMS) of ∼0.3 nm; b) Nb:$SrTiO_3(001)/SrTiO_3$ membrane after the transfer onto the Nb:$SrTiO_3(001)$ substrate, with a RSM of ∼1.3 nm.

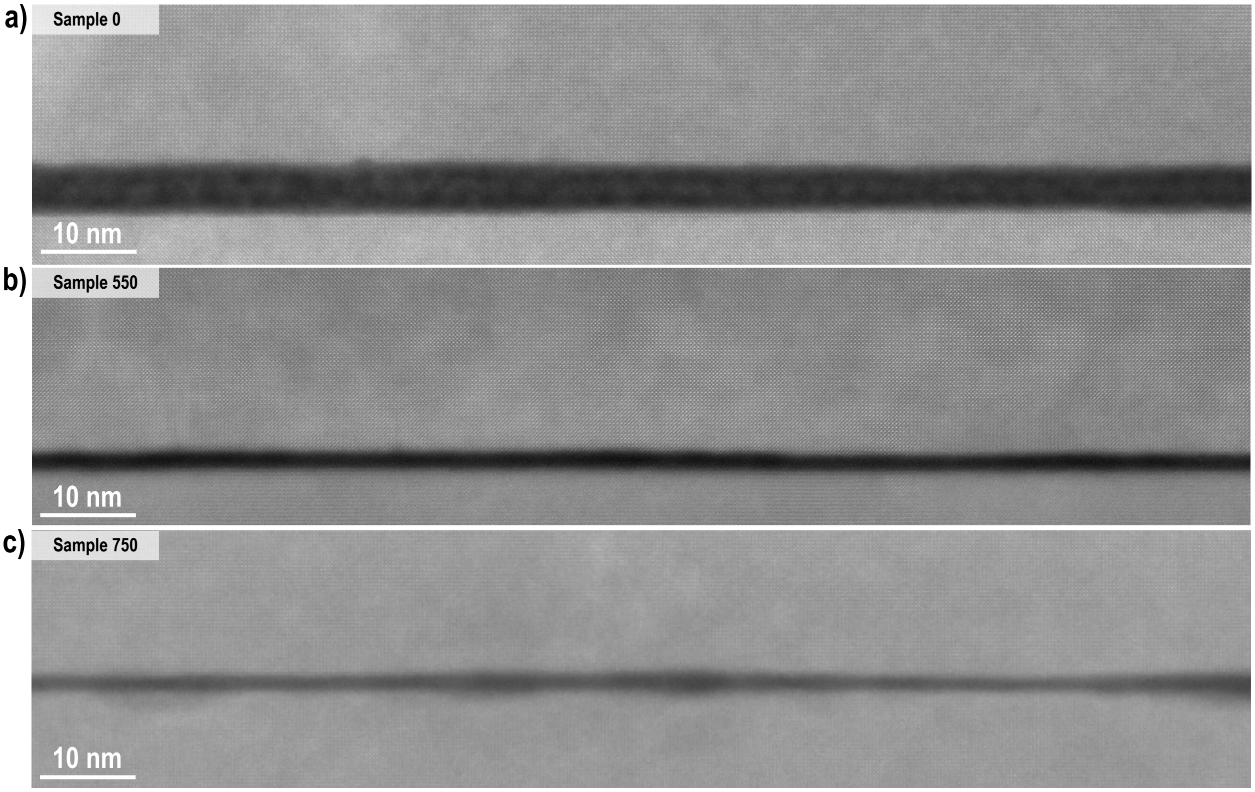

Figure S3: Large area cross-section HAADF STEM images of: a) Sample 0; b) Sample 550; c) Sample 750. A noticeable reduction of the interface gap width is observed after the thermal annealing.

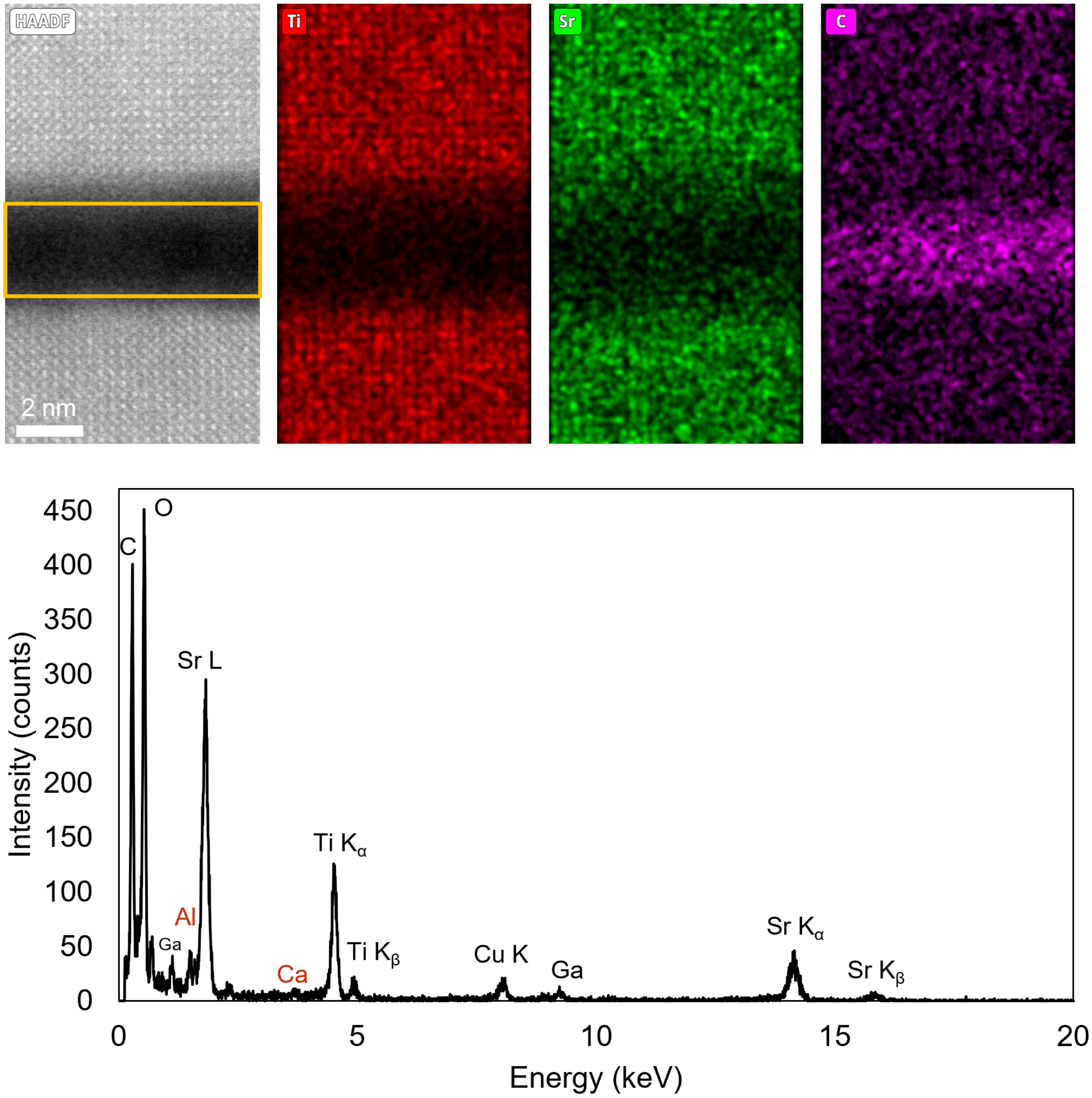

Figure S4: STEM-EDXS analysis of Sample 0 showing presence of Sr and Ti within the interface gap, along with some contaminant elements: C (contamination from contact with air); Al (∼2 at.% – residual from $Sr_3Al_2O_6$); Ga (from focused ion beam milling sample preparation); and Ca (∼1 at.% – residual from the lift-off process).

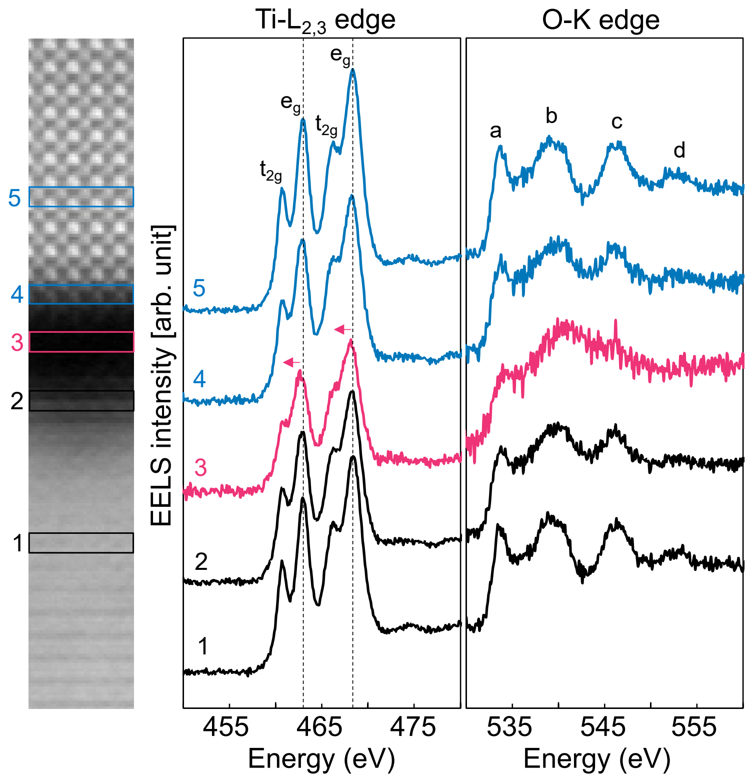

Figure S5: STEM-EELS analysis of Sample 550, from left to right: HAADF cross-section image of $SrTiO_3$ membrane on Nb:$SrTiO_3$(001) substrate after annealing at 550 °C for 1 h; Ti-$L_{2,3}$ and O-K edges extracted from #1 Nb:$SrTiO_3$(001) substrate, #2 Nb:$SrTiO_3$(001) substrate near the bottom interface, #3 center of the gap, #4 $SrTiO_3$ membrane near the top interface, #5 $SrTiO_3$ membrane. The Ti-$L_{2,3}$ edge in spectrum #3 shows some indication of splitting of the L_3 and L_2 peaks, with a slight left shift compared to the substrate spectra, revealing that the Ti begins to "re-gain" its 4+ valence. Features also start to emerge in the O-K edge, as compared to the as-transferred gap spectrum in Figure 2. Note that the HAADF image is cropped from the full width of the original mapped area.

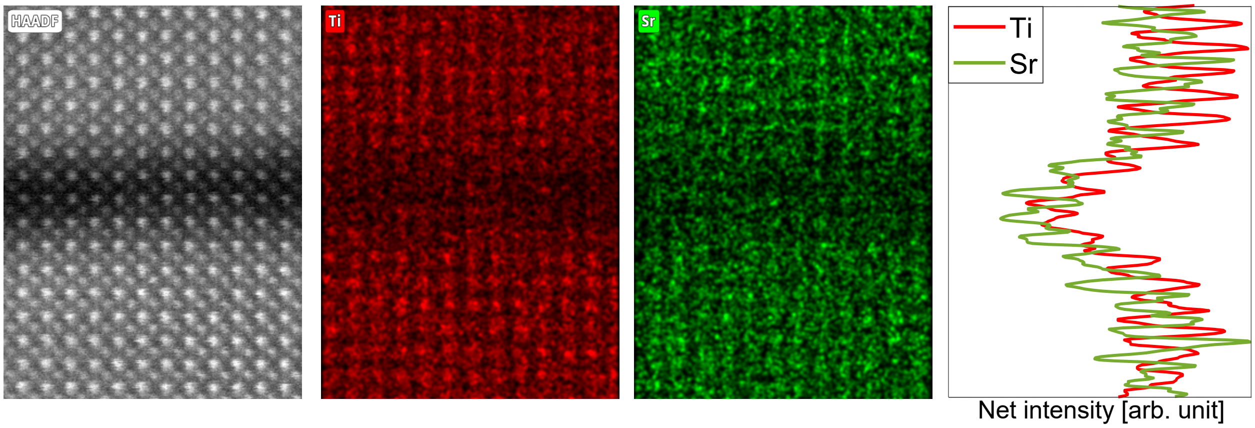

Figure S6: EDXS analysis of Sample 750 presented in Figure 3c, integrating all spectral counts after 311 frames: final HAADF cross-section image of the reconstructed interface of Sample 750 (left); Sr and Ti net count maps (middle); d) integrated intensity line profiles of Sr and Ti across the reconstructed gap interface (right).

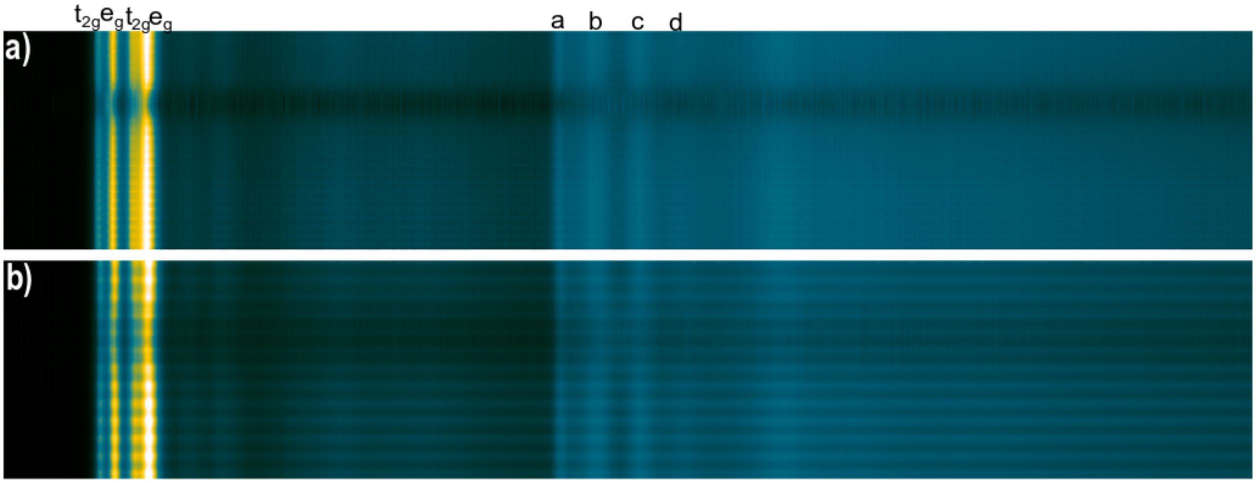

Figure S7: EEL spectra after background subtraction projected along a line in the out-of-plane direction, from before and after the local EDXS e-beam irradiation of Sample 750 that is discussed in Figure 4: a) before EDXS acquisition; b) after EDXS acquisition. Both the increase in fine structure details of Ti-$L_{2,3}$ and O-K edges and the much more defined spatial modulations in the gap from e-beam exposure are clearly discerned with this comparison.

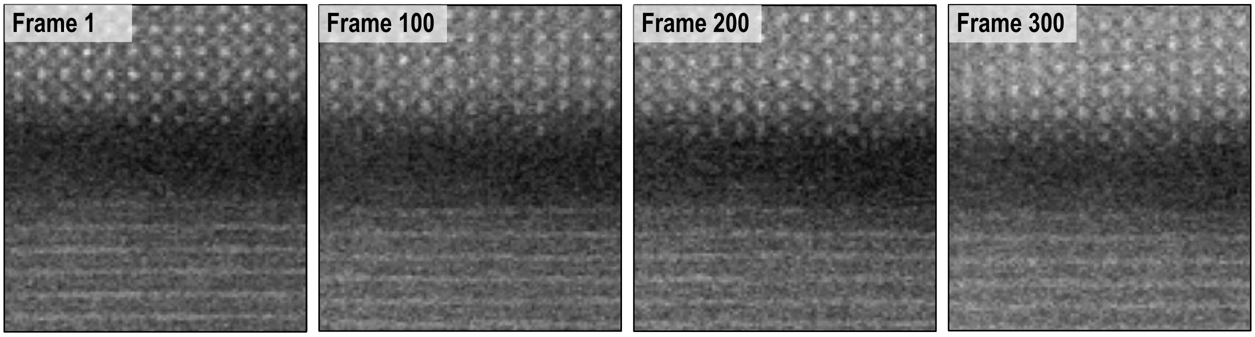

Figure S8: STEM HAADF frames from an EDXS acquisition of a fourth sample that was annealed at 750 °C for 3 h, using a lower beam current of 100 pA, that corresponds to an electron flux of $\sim 10^{10}$ e^{-} Å^{-2} s^{-1}. No evolution of the nanostructure within the interface gap is observed between the frames. Not that the slight, cloudy increase in contrast by the last frame corresponds to typical contamination of the sample during the e-beam raster scanning.

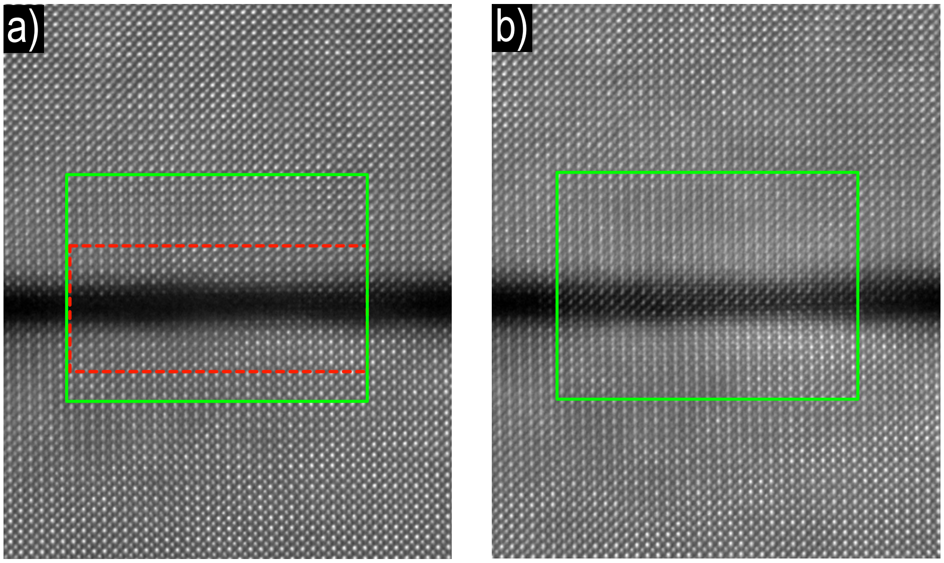

Figure S9: lower magnification HAADF STEM images of Sample 750: a) before local e-beam raster scan; b) after e-beam raster scan (using a "focus window" in DigitalMicrograph). The red box indicates the region that was raster scanned. From inspecting the HAADF image in Figure S9b, it is clear that the new crystalline structure in the interface gap is strongly localized to the raster-scanned region. Further, within the green box, the membrane and substrate crystal structures appear to twist into a new configuration that differs from the surrounding area not subjected to scanning, indicating the creation of local lattice distortions up to a depth of 7–10 unit cells into the pre-existing crystals.

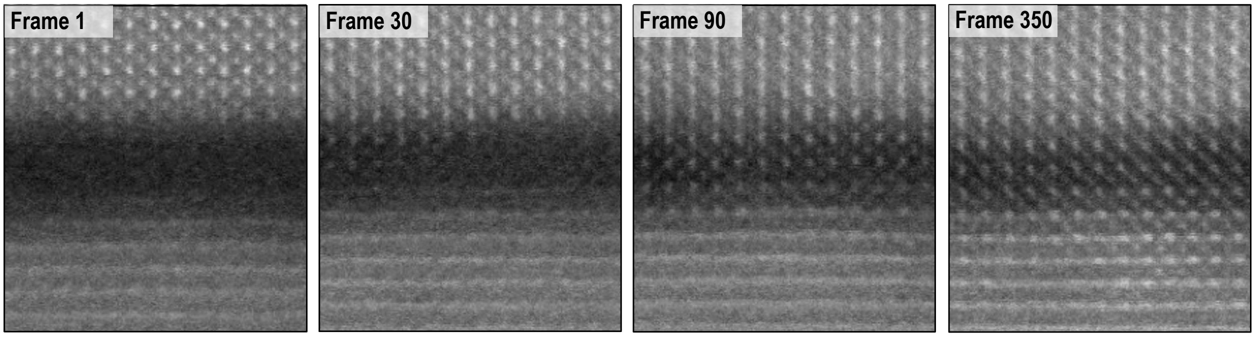

Figure S10: Impact of a larger misalignment between $SrTiO_3$ membrane and Nb:$SrTiO_3$(001) substrate on a fourth sample, annealed at 750 °C for 3 h, having a misalignment between membrane and substrate of ∼4°. Frames are shown from a local EDXS mapping acquisition using the standard e-beam current of 250 pA. By Frame 90, the ∼0.9 nm gap has been filled with new crystalline structure. By Frame 350, the substrate is showing structural realignment up to a depth of ∼3 unit cells.

References

(1) Lucas, G.; Burdet, P.; Cantoni, M.; Hébert, C. Multivariate statistical analysis as a tool for the segmentation of 3D spectral data. *Micron* **2013**, *52-53*, 49–56.
